# Bond strength of surface-treated novel high translucent zirconia to enamel

**DOI:** 10.1080/26415275.2019.1684200

**Published:** 2019-11-04

**Authors:** Martin Ågren, Wen Kou, Margareta Molin Thorén

**Affiliations:** a Prosthodontic Specialist Clinic, Public Dental Service of Västerbotten, Region Västerbotten, Umeå, Sweden;; b Dental Material Science, Department of Odontology, Faculty of Medicine, Umeå University, Umeå, Sweden;; c Prosthetic Dentistry, Department of Odontology, Faculty of Medicine, Umeå University, Umeå, Sweden

**Keywords:** Shear strength, zirconium dioxide, dental bonding, dental veneers

## Abstract

**Aim:** The aim of the present study was to evaluate the shear bond strength of zirconia, stabilised with 5% yttria, luted to enamel and to evaluate the fracture pattern at loss of retention.

**Methods:** A total of 53 test specimen were manufactured from two partially stabilised zirconia materials, Zirkonzahn Prettau Anterior (ZPA) (*n* = 16) and Whitepeaks CopraSmile Symphony 5 layer (WCS) (*n* = 18), and a lithium disilicate (Ivoclar e.Max Press) (*n* = 19) acting as control. All test specimens were cemented to human enamel with Variolink Esthetic DC and then subjected to a shear bond strength test. Fracture and surface analysis were performed using light and scanning electron microscope.

**Results:** No significant differences in shear bond strength were detected when analysing the three groups. Dividing them according to the fracture pattern significant difference in shear bond strength between the two zirconia groups could be seen analysing test bodies with failure of adhesion to the test body, but not to enamel. The ZPA had higher shear bond strength (23.68 MPa) than WCS (13.00 MPa). No significant differences were seen compared to the control group (19.02 MPa).

**Conclusion:** Partially stabilised zirconia shows potential as a material to be used where macro mechanical bonding is not possible, although this study does not reveal how or if the bonding deteriorates over time.

## Statement of significance

As the use of the ceramic zirconia in dentistry is increasingly popular, modifications to enhance aesthetics of the ceramic have broadened the indications for its use. Although, more toothlike in the optical properties than previous generations of zirconia, the material is still not etchable and thus cannot be adhesively bonded to the tooth surface. As the preparation for dental veneers does not offer macromechanical bonding, a strong micromechanical or adhesive bonding is crucial. This study aimed to investigate whether high translucent zirconia could be luted to a flat enamel surface, with similar strength as traditional glass-ceramic dental veneer materials. The results showed that some zirconia may be luted at the same shear bond strength as traditional materials.

## 1. Introduction

Dental laminate veneers are used to restore aesthetics, as well as minor tooth substance loss, and have been in use since the concept was introduced in 1983 [1]. A key factor of veneers is the ability to bond to the tooth substance, thereby reinforcing the strength. Etchable ceramics, which can be adhesively bonded to enamel includes feldspathic porcelain and glass ceramics, such as leucite enhanced or lithium disilicate based [2,3].

The disadvantage of traditional zirconia has been its relative opaque nature and the lack of etchability, a contraindication for using it as a laminate [3,4]. The addition of alumina which has a different refraction of incoming light compared to zirconia makes the ceramic to appear opaque [5]. To increase translucency the alumina content has been decreased from 0.25 wt% to 0.05 wt%, resulting in a more translucent 3Y-TZP, but also a material more susceptible to low-temperature degradation [5,6]. This material, often called translucent zirconia, has significantly higher fracture strength compared to lithium disilicate [7]. Even though it is often referred to as translucent zirconia, 3Y-TZP needs to be thinner than 0.5 mm to be predominantly translucent and not opaque [8].

To further increase the translucency and thus making the optical properties more tooth-like, it is possible to increase the part of cubic zirconia. The third generation of zirconia has an increased amount of yttria added to the formula, increasing the amount from 3 mol% to 5 mol%, thus called 5Y-PSZ, making a mix of tetragonal and cubic phase [9,10]. If the yttria content would be increased to 8 mol% it would make the zirconia truly fully stabilised [11]. In comparison to the tetragonal phase prevalent in standard 3Y-TZP, the cubic phase does not phase transform and thus do not exercise a self-healing nature [9,12]. Cubic zirconia is isotropic, has larger crystals, decreasing the amount of times the light is scattered, making it appear more translucent [13]. The lack of phase transformation of the cubic zirconia impairs the self-healing properties, and thus the 5Y-PSZ has lower flexural strength, approximately 600 MPa compared to 1000 MPa in 3Y-TZP [9,12]. The addition of colouring liquids does not seem to make a significant impact on the flexural strength of 3Y-TZP, but increases the flexural strength of 5Y-PSZ [11].

To achieve adhesion between ceramic and tooth, regardless of being a glass-ceramic or zirconia, the luting agent needs to bond the two different substrates together. In silica-based glass-ceramic through a combination of micro-mechanical retention and chemical bonding. By etching a glass-ceramic surface, it will present a surface of micro retention, with porosities where into the bonding agent can flow and interlock, and a chemical bonding through silanisation of the ceramic surface. By using a luting agent that provides a thin layer and a strong interlock it also strengthens the mechanical properties of the glass-ceramic [14–16]. Compare this to an oxide ceramic sandblasted surface where the surface will be rough, but without the small porosities that create micro retention [17,18].

Oxide ceramics, compared to glass ceramics, are not etchable, instead the surface must be modified using other methods. There is no universal way of achieving a bondable zirconia surface [19]. One way is by airborne particle abrasion, also named sandblasting. The sandblasting creates a rougher surface, thus increasing both the surface area and surface energy, creating undercuts and allowing the luting agent to wet the inner surface of the restoration, creating a micromechanical interlock which is significantly stronger than an unmodified surface. Depending on the distance and air pressure of the sandblasting, as well of the particle size of the abrasive, the blasted surface will be modified differently. Another way is silica-coating, blasting the surface with silica-coated alumina particles which gives an etchable inner surface of the restoration [20]. The term luting agent in this study is interchangeable with the term cement [21].

The loss of a veneer is due to a fracture. Fractures in materials can be divided into two general groups, adhesive or cohesive fractures. The adhesive fracture is a fracture where two different materials separates at bond level and the cohesive fracture is when a part of a material is separated from itself, in this case, the adhesive fracture is when the ceramic would be separated from the enamel and the cohesive fracture is when the fracture is within the ceramic alone or tooth substance alone [22].

Some of the producers of high translucent 5Y-PSZ include veneers within the range of application. As the preparation technique for veneers provides little or no macro mechanical bonding opportunities, the importance of high micro mechanical or chemical bond strength to enamel cannot be stressed enough [23,24].

As veneers traditionally do not rely upon macro mechanical bonding [25], it is important that there is knowledge whether high translucent zirconia veneers truly can be cemented with reliable adhesion to enamel or not.

### 1.1. Aim

The aims of the present study were to evaluate the shear bond strength of 5Y-PSZ luted to enamel and to evaluate the fracture pattern at loss of retention. The null hypothesis is that there are no differences in shear bond strength, nor in fracture pattern, between 5Y-PSZ and the control.

## 2. Material and methods

### 2.1. Dental ceramics

The investigated 5Y-PSZ materials were Prettau Anterior (ZPA) (Zirkonzahn GmbH, Gais, Italy) and CopraSmile Symphony 5-layer (WCS) (Whitepeaks Dental Solutions GmbH & Co. KG, Wesel, Germany). IPS e.Max Press (Ivoclar Vivadent, Schaan, Liechtenstein), a lithium disilicate glass-ceramic was used as a control due to its etchability. All ceramic specimens were produced in the Vita A2 shade, with no additional painting. The ceramic cylinders were milled replicating the inner area of the Bonding Mould Inserts (Ultradent Products Inc., South Jordan, UT, USA) with a diameter of 2.38 mm. The diameter of each ceramic cylinder was controlled using a calliper (500-161 U Mitutoyo UK Ltd, Hampshire, UK). The height of the cylinders was 2 mm. To comply with ISO 29022:2013 at least 15 test bodies were needed from each group [26]. The dental technicians were instructed to manufacture more than 15 test bodies, but not more than 20 if the raw material were sufficient enough. The total number of test bodies was for ZPA 16, WCS 18 and for the control 19, the number fitting into standard discs and everyday production at the dental laboratories involved.

### 2.2. Pre-treatment of ceramics

All ceramics were treated according to manufacturers’ instructions for producing veneers. The WCS were treated with CopraLiSi Connect (Whitepeaks Dental Solutions GmbH & Co. KG, Wesel, Germany), a spray coating the surface of the zirconia prior to sintering with the glass-ceramic lithium silicate, thus giving it etchable properties. After sintering the WCS was etched using 5% hydrofluoric acid (IPS Ceramic Etching Gel, Ivoclar Vivadent, Schaan, Liechtenstein) for 20 s. The ZPA were aluminium oxide blasted (110 µm at 3.5 bars) for approximately 2-3 s at a distance of two centimetres. IPS e.max CAD was etched according to the same protocol as the WCS.

### 2.3. Teeth

Extracted human teeth were collected at the Department of Oral and Maxillofacial Surgery at the University Hospital of Umeå, and stored in purified water grade 1, according to ISO 3696:1987 [27], for 1 to 6 months before testing. The teeth were embedded in epoxy resin. The EpoFix Resin mixed with EpoFix hardener (Struers A/S, Ballerup, Denmark) were poured into moulds with a diameter of 25 mm, SamplKup (Buehler, Lake Bluff, IL, USA) or FixiForm (Struers A/S, Ballerup, Denmark). The resin cylinders were polished flat with CarbiMet SiC abrasive paper (Buehler, Lake Bluff, IL, USA) grit P400, during continuous water flow using a Labopol 21 (Struers Inc, Cleveland, OH, USA). The exposed enamel was either at the buccal, lingual or approximal surfaces. When applying force during the shear bond test the roots where orientated upwards in the opposite direction of the applied force.

### 2.4. Luting agent

The enamel surface was etched with 35% Ultra-Etch phosphoric acid (Ultradent Products Inc., South Jordan, UT, USA) for 20 s, then adhesive was applied onto the enamel surface using Adhese Universal VivaPen (Ivoclar Vivadent, Schaan, Liechtenstein). The adhesive was rubbed onto the enamel surface for 20 s and excess adhesive was removed through airstream and then light-cured with a VALO V34277 (Ultradent Products Inc., South Jordan, UT, USA) for 10 s. After rinsing with purified water, the test specimens were treated with the primer Monobond Plus (Ivoclar Vivadent, Schaan, Liechtenstein) applied for 60 s. Excessive primer was removed by air stream during 5 s. As there are no written recommendations of luting agent for neither Prettau Anterior, nor CopraSmile contact was made with representatives of ZirkonZahn and Whitepeaks. The representatives assured that their products could be cemented using the same method as for the IPS e.Max, which has a recommendation of Variolink Esthetic DC (Ivoclar Vivadent, Schaan, Liechtenstein). The ceramic cylinders were cemented during manual pressure applied for at least 10 s prior to light-curing. Excessive cement was removed using a single-use brush prior to curing and then the cement was light-cured for 40 under continuous manual pressure. The specimens were submerged in water stored at 37 °C for 24 h prior to the analysation of shear bond strength. Before submitting them to the shear bond test, remaining excessive cement was removed using a razor blade.

### 2.5. Method

The procedure was according to ISO 29022:2013 [26] though with some modifications as the ISO standard is mainly written for comparing dental composite adhesion to teeth; the flat grinded teeth only exposed enamel and not dentine, the test specimens were ceramic and not composites. The diameter of the ceramic cylinders was checked by using a calliper (500-161 U Mitutoyo UK Ltd, Hampshire, UK). Any deviation in diameter was compensated in the calculation of pressure withstand before fracture. The specimens were pre-treated and cemented similar to veneers according to instructions from the manufacturer. The shear test was performed using UltraTester (Ultradent Products Inc., South Jordan, UT, USA) with a notched crosshead at a speed of 1.0 mm/min. Failure, loss of retention of the test specimen, prior to loading was recorded as 0 MPa in accordance with ISO 29022:2013 [26].

### 2.6. Fracture and surface analysis

The fractures were analysed using a light microscope (Carton Optical Siam Co., Ltd, Pathum Thani, Thailand) at 30 times magnification. The fractures were categorised to if they were predominantly cohesive, adhesive to enamel or adhesive to the test body. Further to investigate if any cement remnants were left on the surface, two specimens from each group were investigated by a Carl Zeiss Evo SEM (Carl Zeiss AG, Oberkochen, Germany) scanning electron microscope (SEM) and the surface of the lithium silicate-coated zirconia analysed through energy-dispersive X-ray spectroscopy (EDS) to detect if any lithium or silicon was observable.

### 2.7. Study variables

The outcome variables in the present study are MPa.

### 2.8. Statistical methods

The statistical analyses were performed using independent two-sample *t*-test, ANOVA and Levene’s test at *p* < .05. Basic data analyses were performed with IBM SPSS Statistics version 25.0 (IBM, Armonk, USA).

## 3. Results

When comparing the material groups, not dividing according to fracture type, no significant difference was seen between the groups (Table 1). In the ZPA group the lowest shear bond value was 11.1 MPa and the highest 36.7 MPa, in the WCS group the lowest shear bond value, excluding failure prior to loading, was 4.4 MPa and the highest 43.0 MPa. In the control group, the lowest shear bond strength was 10.1 MPa and the highest 34.5 MPa. Mean values with SD were for the ZPA group 21.60 ± 7.32 MPa, the WCS group 16.56 ± 11.09 MPa and control group 18.60 ± 5.86 MPa. No significant difference in shear bond strength was seen between the different material groups (Table 1). One specimen in the WCS group fractured prior to loading, the WCS group showed both highest and lowest shear bond strength, thus had the widest spread of results.

**Table 1.** Difference in shear bond strength. Lithium disilicate e.Max (Control), Zirkonzahn Prettau Anterior (ZPA), Whitepeaks CopraSmile (WCS).

Material	Mean difference (MPa)	95% confidence interval		Sig.
ZPA				
Control	3.36	−3.68	10.41	0.729
WCS	5.40	−1.74	12.53	0.201
WCS				
Control	−2.03	−8.86	4.80	1.000
ZPA	−5.40	−12.53	1.74	0.201

**Figure 1.**
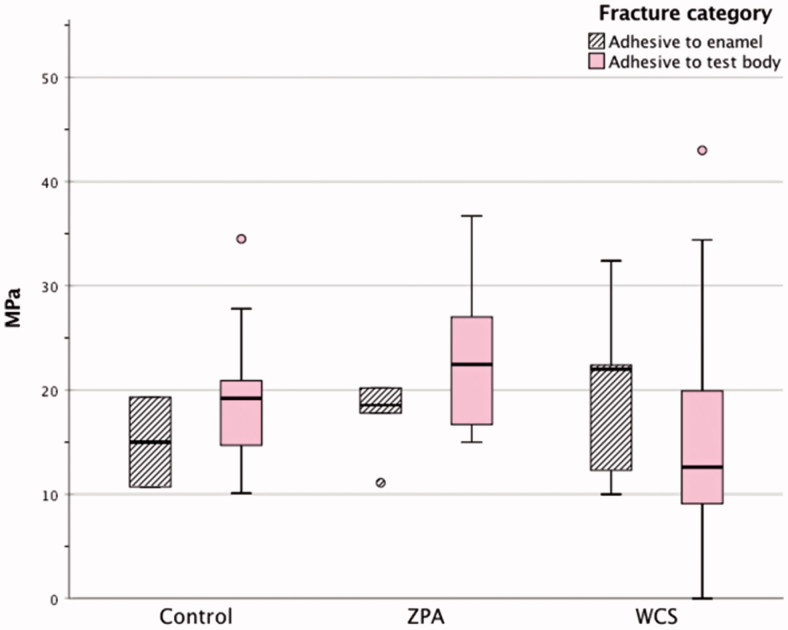
Distribution of shear bond strength according to test groups and fracture type, Lithium disilicate IPS e.Max Press (Control), Zirkonzahn Prettau Anterior (ZPA), Whitepeaks CopraSmile (WCS). Midline in box denotes median value, bottom of box the 25th percentile, top of the box 75th percentile. The T-bars show minimum or maximum values up to 1.5 of the box heights. The circles denote extreme values.

When separating the material groups by type of fracture, in the ZPA group 6 specimen fractured at enamel junction (mean of 19.08 MPa), whereas 10 fractured at test body junction (mean of 23.68 MPa). In the WCS group 5 specimens fractured at enamel junction (mean 19.82 MPa) and 13 at test body junction (mean 15.31 MPa). In the control group 2 specimens fractured at the enamel junction (mean 15.00 MPa) and 17 at the test body junction (mean 19.02 MPa) (Figure 1). Performing an ANOVA analysis and Tukey’s *post hoc* comparing the different materials, no significance is prevalent at fractures adhesive to enamel (*p* .489) whereas fractures at test body revealed a significant difference between material groups at *p* .007. The *post hoc* analysis showed a significant difference in shear bond strength between the ZPA and WCS (*p* .005), all other differences being insignificant (Table 2).

**Table 2.** Difference in shear bond strength when fractured at test body. Lithium disilicate e.Max (Control), Zirkonzahn Prettau Anterior (ZPA), Whitepeaks CopraSmile (WCS).

Material	Mean difference (MPa)	95% confidence interval		Sig.
ZPA				
Control	4.66	−2.58	11.90	0.270
WCS	10.68	2.90	18.60	0.005
WCS				
Control	−6.02	−12.87	0.83	0.095
ZPA	−10.68	−18.46	−2.90	0.005

**Figure 2.**
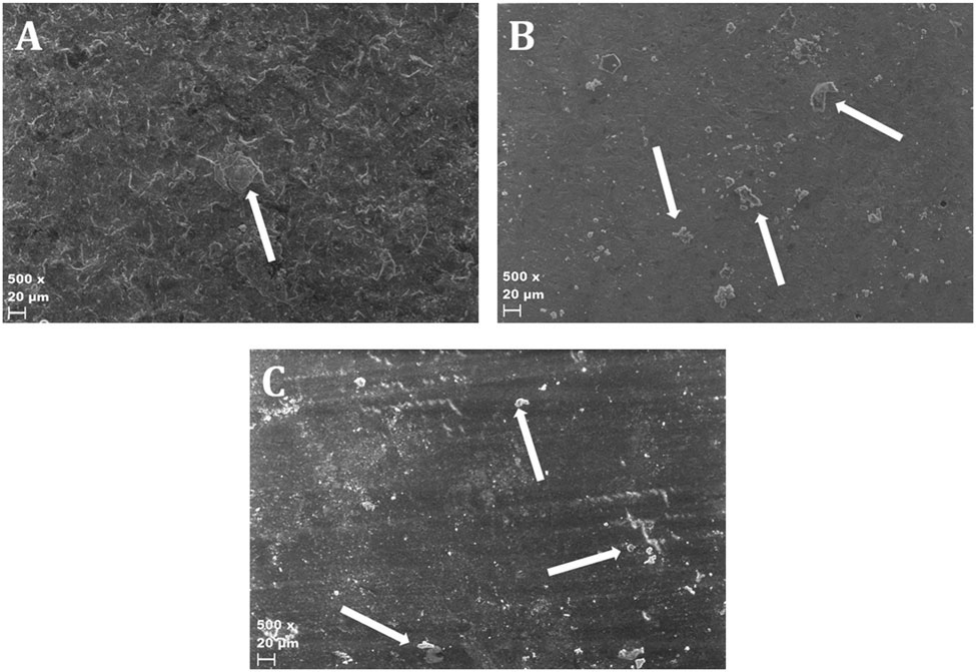
Scanning electron microscope images illustrating the topography of the surface. A = Control (IPS e.Max Press). B = Whitepeaks CopraSmile. C = Zirkonzahn Prettau Anterior. Debris on the surface is remnants of the cement. Arrows indicate examples of debris.

Ocular inspection of the specimen revealed a very smooth surface of the WCS specimens, even after etching. As most fractures occurred between cement and test body some were sent for SEM imaging. The SEM images at 500 times magnification revealed a smooth surface in both the WCS and ZPA specimen compared to the control group (Figure 2), a difference also noticeable through ocular inspection. The surface of all test bodies with adhesive fracture at test body after shear bond test, were relatively homogenous without larger plaques of luting agent residue.

The EDS analysis of the coated WCS revealed that there was no zirconia on the surface, but a high amount of silicon (Table 3). The surface of the control was rough, whereas the WCS had an even smooth surface, the ZPA showed a smooth surface with shallow repetitive grooves (Figure 2).

**Table 3.** Energy-dispersive X-ray spectroscopy analysis of the coated surface of Whitepeaks CopraSmile after fracture testing.

Element	Wt%	Atomic %
C	11.48	17.97
O	50.28	59.11
Na	0.79	0.65
Al	0.58	0.4
Si	30.4	20.36
Cl	0.89	0.47
Nb	2.51	0.51
Ag	3.08	0.54

## 4. Discussion

The aim of this study was to evaluate the shear bond strength of 5Y-PSZ to enamel, the fracture pattern of the luting agent and in extension the possible use of 5Y-PSZ as a dental laminate.

According to Ivoclar Vivadent’s scientific documentation, shear bond strength to enamel of Variolink Esthetic DC, in combination with Adhese Universal, using the UltraTester, is in the vicinity of 20 MPa. This is in accordance with shear bond tests of the Variolink brand of luting system [28–30] although different results are expected as different methods of analysing shear bond strength exists [31]. Shear bond strength results around 20 MPa would be around the upper limit of the luting agent.

The adhesive used, Adhese Universal, contains methacryloyloxydecyl-dihydrogen phosphate (MDP), which has shown the potential of both initial high bond strength as well as longterm bond strength through a reaction between hydroxyl groups on the zirconia surface and phosphoryl groups in the MDP containing bonding. The shear bond strength results from 5Y-PSZ, a zirconia, were in some occasions similar to, or exceeding, the results of the control group, which may be a result of the bonding between the zirconia and the MDP, as studies where primers not containing MDP have been used yielded in significantly lower bond strength [32,33].

Fractures that are due to lack of bond strength at the enamel luting agent junction does not reveal the shear bond strength to the zirconia, as the bonding of the luting agent has not failed to the 5Y-PSZ, but to the enamel. Having a non-significant result at fractures of the adhesion to enamel shows that the adhesion of the luting agent to the enamel is not significantly affected by the restorative material that it is luted against.

The fracture analysis used did not reveal where the fractures were initiated, only if there remained cement remnants on the test specimen or not. As the *t*-test did not reveal any significant differences between the fracture groups within each material group, the results were divided according to fracture type. There is a significant difference between the two 5Y-PSZ groups ZPA and WCS when the luting adhesion failed at the test body. This can be interpreted as the shear bond strength of the material. As the adhesion of the luting agent to the tooth is already well established [1,34,35], measuring failures between test body and luting agent can be interpreted as the shear bond strength of the material, not a measurement of the shear bond strength between the luting agent and the enamel. The low number of fractures at the enamel in the control group could be interpreted as a verification of this statement.

The overall wider span of the shear bond strength in WCS may be a sign of the technical sensitivity of the coating pre-treatment. The pre-treatment consisted of a lithium silicate spray which, according to Whitepeaks, would enable the surface to be etched and adhesively cemented. The method of applying an etchable inner surface of the zirconia, to be able to adhesively lute it to the tooth has been tried previously with varying results [36–44]. Thus, a comparison of shear bond strength between a coated and an uncoated WCS would be interesting. As seen in the SEM, the coating of the WCS creates a very smooth surface, decreasing the possibilities of micromechanical bonding. Although coating of the zirconia with etchable ceramic might present an opportunity for chemical bonding it may decrease the overall shear bond strength, and also possibly total strength of the core, as it adds one more step in producing the final product, and also one more junction, that between the zirconia and the added glass-ceramic where bonding might fail. If successful, a chemical bonding due to an etchable surface may prove more long-lasting and more durable to loading in comparison to the micromechanical bonding [4,39].

The fact that no lithium was detected on the WCS surface may be explained by a limit in the EDS analysis when trying to detect elements with a low atomic number [45]. As the technical data of WCS provided from Whitepeaks does not contain silicon nor lithium, the amount of silicon detected (Table 3) reveals that the surface composition has been modified by the LiSi-spray, but the SEM image (Figure 2) reveals that the surface does not provide micro retention after hydrofluoric etching. The reason to why the LiSi-spray does not increase the shear bond strength might be to the composition or application method of the spray.

The sand blasted surface of the ZPA is, through SEM imaging, seemingly as smooth as the WCS, but with repetitive grooves (Figure 2). Sand blasting itself does not increase the micromechanical bonding surface after sintering, but may work as a way of cleaning the zirconia surface as alumina sand blasting improves shear bond strength compared to an as-sintered surface [39]. Sandblasting exclusively prior to sintering have been showed to increase the surface roughness, but with similar results of shear bond strength as when sandblasting after sintering [46]. The grooves seen in SEM are presumably produced by the milling of the surface. They may contribute to increase the surface area and thus increasing the strength of bonding [47]. Micro machining of zirconia is possible [48] and could be a way to increase surface area and retention [47].

The test results cannot be directly compared to other shear bond strength tests where a different test method has been used, as the values (MPa) will differ within the same material applied to different methods of analysing shear bond strength [31]. A uniform standard for testing shear bond strength to ceramic restorative materials is currently lacking. A suggested way of testing this is by replacing the tooth in the ISO 29022:2013 [26] with the ceramic specimen, applying the luting agent, and bonding a composite on top using the standard mould for the Ultratester. This is just a slight modification of the method used to test shear bond strength in composites, and therefore no separate equipment is needed to modify it to test zirconia or other materials for shear bond strength.

As there was no significant difference between the different groups one can conclude that within the limitations of this study the initial micromechanical bonding is as effective as the adhesive bonding and thus 5Y-PSZ cannot be excluded, at this stage, as suitable for restorations without macro mechanical retention. An increase of test specimens, or by eliminating the enamel, would increase the power of a future test. This test does not reveal if the retention to the 5Y-PSZ deteriorates over time and if there would be a significant difference compared to the control.

## 5. Conclusions

5Y-PSZ shows potential to be used in preparations not made for macro mechanical bonding, although the pre-treatment of the 5Y-PSZ differ between manufacturers and may have a great impact on the retention over time. There were inconsistencies if there were significant differences in shear bond strength between zirconia and the control group, therefore the null hypothesis is partly retained.
